# From the Andes to the Apennines: Rise and Fall of a Free-Ranging Population of Feral Llamas

**DOI:** 10.3390/ani11030857

**Published:** 2021-03-18

**Authors:** Carlo Gargioni, Andrea Monaco, Gentile Francesco Ficetola, Lorenzo Lazzeri, Emiliano Mori

**Affiliations:** 1 Department of Environmental Science and Policy, Università degli Studi di Milano, Via Celoria 26, 20133 Milan, Italy; carlo.gargioni@gmail.com (C.G.); francesco.ficetola@unimi.it (G.F.F.); 2 ISPRA Institute for Environmental Protection and Research, Via Vitaliano Brancati 48, 00144 Rome, Italy; andrea.monaco@isprambiente.it; 3 Research Unit of Behavioural Ecology, Ethology and Wildlife Management, Department of Life Sciences, University of Siena, Via P.A. Mattioli 4, 53100 Siena, Italy; lazzerilorenzo12@gmail.com; 4 Istituto di Ricerca sugli Ecosistemi Terrestri (IRET), Consiglio Nazionale delle Ricerche (CNR), Via Madonna del Piano 10, 50019 Sesto Fiorentino, Italy

**Keywords:** feral species, introduction pathways, *Lama glama*, social perception, unsafe management

## Abstract

**Simple Summary:**

Domestic mammals may become invasive alien species when introduced within natural environments and when they establish reproductive populations. One of the most common pathways of species introduction is represented by intentional or accidental escapes from confined environments, including zoos, farms and enclosures. A feral population of llama *Lama glama* has been present in Central Italy since 2016 after escaping from a zoological garden. In 2020, only three individuals were confirmed to be still present within a 40-hectare area, following a field survey. We carried out questionnaires with the resident human population to determine the local perception and the acceptance of two possible management actions, i.e., direct killing and surgical sterilization. Llamas are quite docile domestic animals, thus local perception was, in general, very positive and also linked to the exotic origin of the species, making llamas a welcome observation and a pleasant surprise. The observed decline of this population may be due to predation by wolves and poaching, together with the lack of suitability of natural environment, which may have prevented llamas establishing an invasive population. In this context, however, individual removal action should be conducted before the population shows a demographic rebound.

**Abstract:**

Since 2016, a feral population of llama *Lama glama* has been present in Central Italy after escaping from a zoological garden and starting to reproduce. We updated demographic status and distribution of this population and investigated societal perception towards the llama presence and management in the area through a standard questionnaire. Field data were collected through direct (transects traveled by car and on foot) and indirect (newspapers, social networks and online platforms) research. The feral population appears to be declining. In July 2020, the population was represented by three individuals (one male and two females), identified also through photoidentification, most likely located within a 40-hectare area. The majority of citizens are aware of the presence of feral llamas and show a positive attitude toward them and a negative one toward management actions. The case of feral llamas in Italy is an evident example of unsafe management of a species which should have kept in a zoo and which, once set free, was able to catalyze the attention of the general public. The decline of this population limits the need of drastic management actions that, given the appreciation expressed by people and press toward these animals, would have been at risk of conflict with the public opinion. Removal action should be rapidly taken, i.e., before any demographic rebound and before the population becomes a stable feature of the local landscape.

## 1. Introduction

Biological invasions are one of the main causes of the current global biodiversity crisis [1,2,3]. Alien species are defined as taxa released outside their native extent of occurrence by direct or indirect human intervention [1,4]. Following this definition, domestic species which establish free-ranging populations in the wild, often defined as “feral species”, should be considered as “alien”, as they would not exist in the wild without human intervention [5,6]. Escapes from zoos and farms represent a common pathway of alien species introduction [7,8,9,10,11,12]. Despite several strict laws [13,14], animal escapes may occur following accidental events, unsafe management (i.e., in absence of preventative measures, such as adequate fencing systems, to prevent escape of captive animals into the wild) or irresponsible pet ownership [15,16,17,18]. All alien species have an impact on the ecosystem where they are introduced, on the economy and/or on human and wildlife health [19,20,21,22]; nevertheless, quantification of impacts often requires time-consuming and expensive fieldwork by experts in biological invasions [23]. Some free-ranging domestic species are among the worst invasive alien species, being able to rapidly expand their range and population densities, as well as strongly affecting native ecosystems [24,25,26,27]. Amongst domestic herbivores, goats and sheep are globally widespread on islands and their impacts were previously assessed [25,28,29,30]. Also, the dromedary *Camelus dromedarius* was introduced to Australia in 1840 and it is quickly expanding, threatening the native vegetation [31,32,33]. As a consequence, early management actions and eradications are highly recommended by the Convention on Biological Diversity and by guidelines of the Invasive Species Specialist Group of the IUCN. However, management activities toward free-ranging domestic species and pets, particularly if they involve a direct action on animals (e.g., direct killing or surgical sterilization), are often unpopular and can be opposed by the public opinion [34,35,36,37,38]. Therefore, human attitudes need to be assessed to avoid protests or to limit negative responses to management actions [39,40,41,42].

The llama, *Lama glama*, is the domestic form of the guanaco *Lama guanicoe* [43], which was domesticated in South America for wool and meat production about 6000–7000 years ago [44,45]. The llama was introduced worldwide as an attraction in parks, farms and zoos [46]. In Central Italy, a self-sustaining population of feral llamas originated from a group of captive animals living in a local zoo (Cavriglia, province of Arezzo, open between 1974 and 2016) since 2016 [46]. Llamas were kept as free-ranging within the zoo borders since 1993 but, after 2016, they were abandoned and started to freely range also in the surroundings of this zoo. In 2017, the population of feral llamas reproduced in the wild and without food provided by humans for the first time. In the same year, the llama population included at least six individuals (four adults and two juveniles) in the proximity of the release area [46]. Potential ecological impacts of feral llamas included overgrazing and competition with native wild ungulates [46]. 

In this work we aimed at (i) updating the demographic and distribution status of this population of free-ranging llamas five years after the zoo closure [47] and (ii) describing the public perception on the presence of llamas and attitudes towards two actions (direct killing or sterilization), often used in biological invasion management programs. Considering that the perception of a species is often related to its visibility [42], and llamas are large-sized ungulates well-adapted to human presence, we predicted that llamas would be highly appreciated in our study area.

## 2. Materials and Methods

### 2.1. Study Area

Our study area (about 9000 hectares) included a hilly area in Central Italy (Chianti hills and Valdarno provinces of Arezzo, Siena and Florence), with two Special Areas of Conservation (SAC) included in a network of nature protection areas within the European Union called Natura2000 (following the Habitats Directive 92/43/EEC and the Bird Directive 79/409/EEC). SACs covered over the 75% of the surveyed area (Special Area of Conservation (SAC): IT5190002 “Monti del Chianti” and IT5180012 “Valle dell’Inferno e Bandella”). The SAC “Monti del Chianti” (about 7500 hectares) is included in the Chianti hills area, mostly covered with deciduous woodlands (*Quercus cerris*, *Quercus pubescens*, *Castanea sativa*: 38%) alternated with vineyards (27%), surrounded by scrublands (11%) and fallows (mostly with *Bromus erectus*: 14%), with rare human settlements (8%) and coniferous woodlands (2%). Wild ungulate assembly includes roe deer *Capreolus capreolus*, wild boar *Sus scrofa* and introduced red deer *Cervus elaphus* and fallow deer *Dama dama*; the only large carnivore in this study area is the grey wolf *Canis lupus*. The former zoological garden of Cavriglia hosts currently a camping area (“Camping Orlando”) and several abandoned buildings and precincts [46]. The SAC “Valle dell’Inferno e Bandella” (about 531 hectares) is also a Regional Natural Reserve and is located along the Arno river (municipalities of Bucine and Terranuova Bracciolini, province of Arezzo). This area is all covered by patches of deciduous woodlands (*Quercus cerris*, *Quercus robur*, *Carpinus betulus*, *Salix* spp. and *Populus* spp.: 45%), wetlands/riverine vegetation (22%), scrublands (8%) and farmlands (23%), with scattered human settlements (2%). The wild ungulate guild of this area includes the wild boar, the roe deer and the fallow deer; furthermore, feral domestic pigs *Sus scrofa domestica* are also reported. 

### 2.2. Data Collection

**Figure 1:**
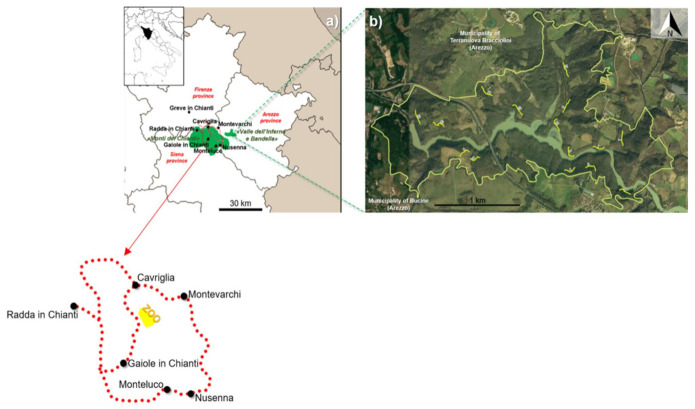
(**a**) Location of the study area (Central Italy, with green areas showing the two Special Areas of Conservation where our survey was conducted). The red dotted line indicated by the red arrow shows the 89 km road traveled each day (yellow polygon = ex Cavriglia zoo park); (**b**) the yellow segments show the 15 paths (200 m each) traveled by foot within the “Valle dell’Inferno and Bandella” Natural Reserve. The continuous yellow line shows the border of the Valle dell’Inferno reserve, where some individuals were observed in 2019 (see results).

In July 2020, we traveled for five consecutive days in the early morning [h06:00–10:30] and in the afternoon [h17:00–21:30], i.e., when llamas are most detectable [46], along an 89-km road in the municipalities of Montevarchi and Cavriglia in the province of Arezzo, as well as Gaiole in Chianti and Radda in Chianti in the province of Siena, to update distribution and demographic status of the feral llama population (Figure 1). We recorded the number of observed individuals, coordinates of each record and estimated age (adult, subadult, young) on the basis of body size [45,46]. The road was traveled by car at a mean speed of 50 km/h. We changed the starting point every day to increase the probability of detecting llamas where present [46].

The traveled road included all the area where llamas were recorded [46] apart from the “Valle dell’Inferno” Natural Reserve. The “Valle dell’Inferno” Natural Reserve does not include paved roads; thus, 15 pathways (200 m each) were traveled by foot once a year in the framework of a monitoring program on wild boar populations in September 2019 and September 2020 to search for signs of llama presence.

To update demographic and distribution status and trends after 2017, i.e., after the previous llama survey [46], further photographic information on distribution of free-ranging llamas were collected through research on social networks (Facebook), video- and photo-sharing websites (i.e., YouTube and Flickr), online newspapers and platforms for naturalistic data collection (i.e., iNaturalist, data downloaded on 3 August 2020). Collected data were validated by contacting observers or photo authors, and by asking them further information on number of observed individuals and precise coordinates. Patterns of coat color allowed individual recognition (photoidentification) across years, apart from monochromatic white individuals (up to 4–5 white individuals in 2016). Again, all the individuals were classified as adult or young according to body size [45,46]. In 2020, all observed llamas showed a unique coat pattern allowing individual identification. To estimate the population size in 2020, we applied a capture-mark-recapture (CMR) protocol based on photoidentification [48]. To assess the annual variation of the distribution of this population, we used occurrences collected in each year between the previous survey in 2017 [46] and 2020. Then, we calculated the Minimum Convex Polygon (MCP) area for each year, which is among the most reliable approaches when data are few [49], encompassing all occurrences of each year with a 50 m wide buffer area [50] using the packages *ade4* [51] e *adehabitatHR* [52] for the software R 3.5.1 [53]. 

### 2.3. Social Perception of Feral Llamas

We assessed social perception in a sample of residents of the municipalities of Greve in Chianti, Radda in Chianti, Gaiole in Chianti, Cavriglia, Montevarchi and Bucine (N = 84) by administering a structured questionnaire (Table S1 in Supplementary Material), to assess the local perception and popularity of llamas and resident opinion toward their removal. We directly surveyed all the citizens in the main towns and villages (Nusenna, Monteluco, Montevarchi, Bucine, Gaiole in Chianti, Osteria della Passera, Radda in Chianti, Cavriglia) we were able to find; the small sample size was due to human movement limitations due to the COVID-19 pandemic outbreak. Further analyses were only carried out on a total of 62 filled questionnaires, as 22 residents did not complete the survey. All surveyed people were over 18 years old and able to autonomously fill the questionnaire, and agreed to participate in this research, with respect to the National and International Italian laws on privacy and sensitive data (DL 196/2003; EU Regulation 2016/679). All questionnaires were implemented on paper and were anonymous and conducted through autocompletion to avoid potential influences by operators. Results were shown as percentages (frequency of occurrences, i.e., total number of each *i-*answer/total number of questionnaires, for each question) in a bar graph.

### 2.4. Media Content Analysis

Social perception was also assessed through a media content analysis, which helped understand the communication environment and public attitudes toward this topic [54]. In September 2020, we carried out research using the Google search engine to screen online newspapers and magazines [55], by using the search terms (in Italian) llama, Chianti, Cavriglia zoo, llama escape, Chianti hills, Valle dell’Inferno, Gaiole in Chianti, Panzano. Articles were divided into three categories with respect to their attitude to the presence of llamas [55]: (1) neutral, where the news was objectively reported, without expressing the journalist opinion; (2) positive, where the news supported the presence of free-ranging llamas; (3) negative, where the news highlighted problems, critics or a clear opposition to the presence of llamas. Scoring was conducted by three authors independently (CG, EM and LL) and validated through a discussion amongst authors.

## 3. Results

### 3.1. Llama Records and Population Range

In our survey (July 2020), we were able to detect only three llama individuals, one male and two females. After the total closure of the Cavriglia Zoological Park, at least 27 occurrences of free-ranging llamas were collected and/or reported on online platforms, social networks and websites of video/photo sharing between 2017 and 2020 (Table 1; Figure S1 in Supplementary Materials). No evidence of presence was recorded in 2020 in the random paths traveled in the SAC “Valle dell’Inferno e Bandella”.

**Figure 2:**
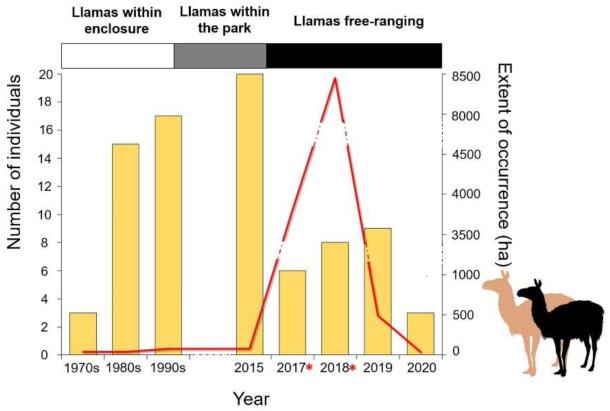
Trends of population size (bars: minimum number of individuals) and extent of occurrence (red line) of the llama population. Population data between 1970 and 2017 were taken from published literature [46]. Llamas lived in an enclosure within the park in the 1970s–1990s (llamas within enclosure), then outside the cage but still within the zoo up to 2016 (llamas within the park) and then outside the zoo park borders. Asterisks mark years with known reproduction events in the wild (i.e., photos of juveniles or suckling cubs). Dashes within the red line reflect dashes in the right *y*-axis.

**Figure 3:**
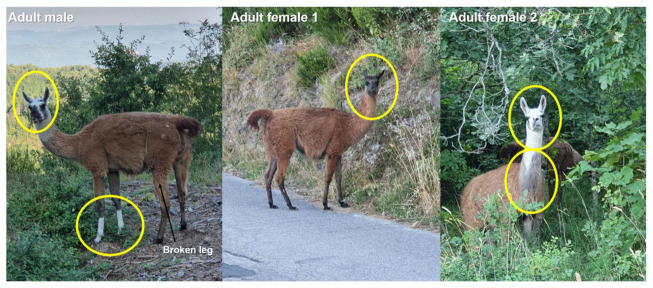
The last three individual llamas observed in January–August 2020 (photo Carlo Gargioni). Circles show individual diagnostic features.

Reproduction in the wild was confirmed in 2017 and 2018, whereas we have no evidence of births in 2019 and 2020 (Figure 2). Llamas showed the broadest distribution range in 2018 (i.e., about 8500 ha), while range sharply declined in 2019 and 2020 (40 ha; Figure 2). At least five or six individual llamas were photographed in 2017 (*n* = 13 photos with a total of 17 llamas), five or six in 2018 (*n* = six photos with a total of nine llamas), four or five in 2019 (*n* = five photos with a total of seven llamas; Figure 2 and Figure S2 in Supplementary Materials). All 26 photos taken in 2020 (and observations anecdotally reported) were shot along the same 4.7 km stretch of road (included in the 89 km road we sampled twice a day, every day). All photos belonged to the same three visually identified individuals, and three was also the maximum number of individuals observed together (Figure 2 and Figure 3). Observations of outer genital organs allowed us to confirm that they were one male and two females. Llamas can live up to 21 years [56], thus the same individuals may have been observed across different years and could be identified across years through fur color patterns. Unfortunately, it was not possible to reliably assess individual sex from downloaded photos of other individuals recorded in previous years.

### 3.2. Social Perception

**Figure 4:**
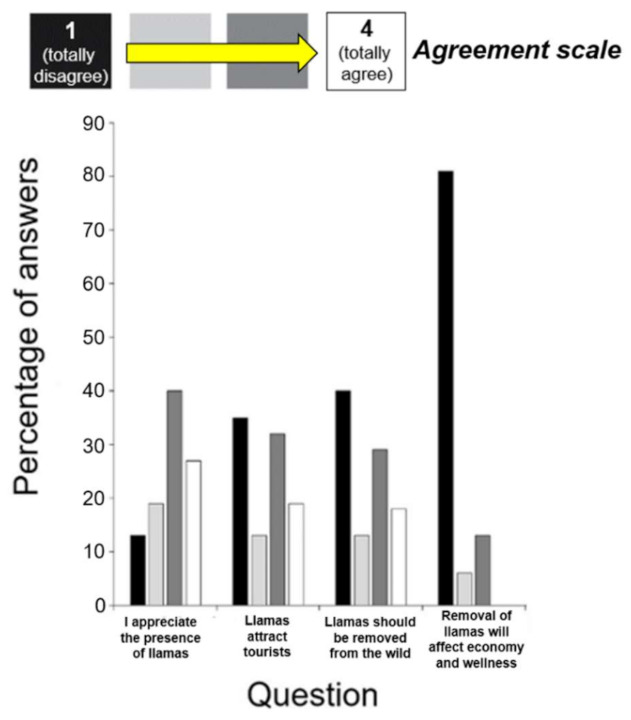
Frequency of answers to the survey on social perception: questions 1–2) social perception; questions 3–4) attitudes toward local removal of llamas.

About 87% of surveyed citizens were aware of the presence of feral llamas in the study area. As for the remaining 13%, most specified spontaneously that they were thinking that free-ranging llamas have gone extinct and two citizens had lived in the study area for less than two years. Most citizens (67%) considered llamas as a potential touristic attraction, therefore resulting in a general positive attitude. Half of the surveyed citizens (53%) were against the removal of llamas to protect native biodiversity, although most of them (81%) also confirmed that this practice would only produce a limited, temporary regret in the resident human population, with no effect on tourism and local economy (Figure 4; Table S2 in Supplementary Material). 

The media content analysis was carried out on 18 newspaper/popular magazine articles (Table S3 in Supplementary Material), eight of which were published in national online newspapers. Overall, 12 (66.7%) articles were classified as positive toward the presence of feral llamas and did not include expert opinion (zoologists, researchers or technicians), whereas only one (with expert opinion, 5.6%) was negative and highlighted that llamas are not a native species. The remaining five articles (27.7%) were neutral, i.e., addressing llamas as alien species without showing problems due to biological invasion, or talking about their local, non-quantified impact on cultivations, thus without expression of positivity or negativity linked to the presence of this species.

## 4. Discussion

In our work, we updated the demographic and distribution trends of the only currently known population of feral llamas in Europe, four years after humans stopped food provision in the zoo borders and three years after the previous assessment [46]. We also collected, for the first time, data on social perception and attitudes toward the species in Italy and possible management options. The number of individuals increased slightly between 1993 and 2016, when llamas were set free to move outside the enclosure (but still confined inside the zoo perimeter), reaching a maximum of 17 individuals [46]. Between 2017 and 2020, the presence of free-ranging llamas was reported not only around the borders of the Zoological Park of Cavriglia, but also in a broad area (about 8.500 ha) encompassing three Tuscan provinces (Arezzo, Siena and Florence) and including the Chianti hills and the “Valle dell’Inferno e Bandella" Natural Reserve. Predation by wolves was reported by several citizens, leading to the death of at least two young individuals. Similarly, at least two cases of poaching occurred in Panzano in Chianti due to local complaints of crop damage. Several other cases of predation and poaching might have triggered and accelerated the population decline of feral llamas. 

Despite the amount of photographic evidence, it was not possible to accurately reconstruct kinship relationships among individuals observed since 2016, also because of the presence of monochromatic white individuals. Furthermore, five white llamas were observed in “Valle dell’Inferno and Bandella” Natural Reserve in 2019, although field work in September 2019 and September 2020 did not confirm their occurrence. Therefore, only three individuals were confirmed in summer 2020, located in a very small area (40 hectares) around Monteluco in the municipality of Gaiole in Chianti (Siena). These individuals included two females (one brown and one brown with white head) and one male (brown with a white head and front legs). One of the hind legs of the male appeared to be injured after a collision with a car in 2019. We feel confident that it is very unlikely that further individuals would not have been detected in our survey, because (i) llamas, particularly adult ones, are confident and well-adapted to human presence and food provision (as supported by the number of photos along paved roads, including selfies; Figure S2 in Supplementary Material), (ii) the area is highly traveled by humans (hunters and mushroom gatherers) who would have probably met further individuals, (iii) there is a high tendency to publish on social networks any encounter with exotic animals, particularly when they are as confident as llamas. Despite the report of at least two reproduction events distant from the release site, we confirmed that the population is still localized, numerically rare and declining (stage III, *sensu* [57]; Category C2-C3 *sensu* [47]). However, it is not possible to exclude a future increase in number and spread [58].

The general positive social perception toward the presence of feral llamas in the study area fulfilled our prediction. This positive perception may have been promoted by the high visibility of these animals [42], as also confirmed by the fact that most residents were aware of their presence. The appreciation of free-ranging llamas finds another explanation in the docile nature, goofy look and confident behavior of these animals (escape distance < 5 m; [46]). The positive feelings were fully reflected by the local and national press, which mostly expressed positive feelings about the presence of these free-ranging animals. Whenever residents expressed negative feelings toward the presence of free-ranging llamas, this was justified through ecological arguments, underlining issues linked to biological invasions [1,4]. Few citizens (*n* = 4) were negative with respect to the presence of llamas in the study area, specifying how the introduction of an ungulate species may represent an additional potential source of crop damage, together with wild boar and deer. Citizen who felt that llamas could represent a tourist attraction discussed that this would happen only if llama–man encounters occurred. In other words, the choice of visiting the Chianti area would not be influenced by the presence of the llamas. Unfortunately, it was not possible to carry out a survey investigating tourist perception due to the very limited tourist pressure in summer 2020 related to the emergency status due to the SARS-CoV-2 pandemic outbreak [59]. Opinions on the removal of llamas from the environment to protect native flora and fauna contrasted one-another. Half of the surveyed people disagreed, arguing that llamas should remain in the area as they do not cause any damage. Citizens in favor of removal of the llamas from the natural environment justified their claim on the basis of ecological principles (species outside their native range) or by referring to the potential risk of collision with vehicles and crop damages. Although no particularly critical elements emerged, e.g., highlighting the urgency of management actions, it should be at this moment that we should plan the removal of the llamas from the wild, e.g., by capturing and translocating them to an appropriate enclosure, since early management has highest probability of success [46]. A population rebound starting from only three remained individuals may seem unlikely. However, several populations of invasive or reintroduced species started with a low number of released individuals and are now composed by a high number of individuals (e.g., grey squirrels *Sciurus carolinensis* introduced to Italy [60], ibex *Capra ibex* reintroduced to the Marmolada massif, where they became extinct [61]).

Considering our data on abundance and distribution of this population and on the species traits (e.g., gregarious behavior, large size, reduced escape distance), removal from the environment appears to be still technically feasible and with a relatively small operational effort. 

## 5. Conclusions

Understanding factors which determine the success of invasive species requires information on both successful and failed introductions. Not publishing the data on failed introductions would cause publication bias, hampering appropriate scientific syntheses. 

Domestic mammals can become invasive alien species when introduced by humans within natural environments. One of the most common pathways of species introduction is represented by intentional or accidental escape from confined environments, including zoos, farms and enclosures. The case of free-ranging llamas in Central Italy was reported as an example of unsafe management of animal species in a confined environment [46]. Conversely, it may now become a case of safe management of a free-ranging alien species if removal action is rapidly taken, i.e., before the population shows a demographic rebound (e.g., due to some uncontacted nucleus) and before it can become a stable feature of the local “landscape” [62]. As a consequence of the latter, tolerance toward the species would be developed, as well as affectivity by local people and tourists, which would make any future decisive intervention very difficult. Although we are aware that only a few alien species become invasive and that this llama population was only composed of very few individuals, we also believe that it is the best time to act for removal following the precautionary principle and the general guidelines for the management of biological invasions. 

After our survey, an agreement between the Tuscany Region, the Municipality of Gaiole in Chianti, the Province of Siena, the Italian Forestry State Body and several associations of hunters and animal rights groups organised the captures of the last free-ranging llamas, to ensure their safety and that of motorists. All the individuals recorded in our work and a newborn (son of one of the two females in Monteluco) were captured with the help of veterinarians and moved to an enclosure at the Wildlife Rescue Center of Semproniano (province of Grosseto).

## Figures and Tables

**Table 1:** Records of encounters of free-ranging llamas (minimum number of individuals) in Monti del Chianti and Valdarno since 2017 (synthesis of our survey and previous sources).

Year	Min. N Individuals	Latitude (°N)	Longitude (°E)	Location	Data Origin
2017	5	43.548432	11.397699	Badia a Coltibuono	[46]; photos by local people
2017	4	43.541079	11.400526	Badiaccia a Montemuro	[46]
2017	1	43.534617	11.420925	Cafaggiolo	[46]
2017	1	43.538865	11.420130	Caiano	[46]
2017	2	43.545886	11.403143	Caiano	[46]
2017	5	43.540687	11.419126	Caiano	[46]
2017	2	43.542622	11.408738	Camping Orlando—Cavriglia	iNaturalist; photos by local people
2017	3	43.539714	11.409792	Cavriglia	Dodaro et al., 2019; photos by local people
2017	3	43.540616	11.413999	Cavriglia	iNaturalist
2017	3	43.544332	11.412823	Cavriglia	[46]
2017	2	43.541926	11.415896	Cavriglia	iNaturalist; photos by local people
2017	3	43.539700	11.418975	Cavriglia	[46]
2017	2	43.541631	11.415181	Cavriglia	[46]
2017	1	43.546706	11.394140	Monte San Michele	[46]
2017	3	43.542724	11.316486	Panzano	[46]; Youtube
2018	2	43.530708	11.422933	Cavriglia	iNaturalist
2018	2	43.471998	11.436921	Gaiole in Chianti	Facebook and Youtube
2018	1	43.428363	11.410002	Osteria della Passera	Photos by local people
2018	2	43.548432	11.397699	Badia a Coltibuono	Facebook; photos by local people/tourists
2018	3	43.488398	11.402336	Radda in Chianti	Photos by local people
2018	4	43.443966	11.508516	Monteluco	YouTube; Facebook
2018	4	43.451299	11.530570	Nusenna	Photos by local people
2019	4	43.451299	11.530570	Nusenna	Photos by local people
2019	1	43.442528	11.453818	Castagnoli	Photos by local people
2019	5	43.519675	11.666618	Valle dell’Inferno e Bandella	iNaturalist
2020	3	43.444639	11.480960	Castellare	Photos by local people/tourists
2020	3	43.444908	11.507084	Monteluco	Our survey

## Data Availability

Used data are included in Supplementary Materials.

## Supplementary Materials

The following are available online at https://www.mdpi.com/2076-2615/11/3/857/s1. Table S1: Questionnaire conducted to test for social perception of feral llamas in Central Italy. Table S2: Results of the standard questionnaire (*n* = 62 citizens, >18 years old, agreed to participate in this research). Agreement was expressed in a four-level scale ranging from 1 (totally disagree) to 4 (totally agree), as in Table S1. Table S3: Results of media content analysis, with link to the analyzed articles. Figure S1: Some photos of llamas observed in 2017–2020 (cf. Table 1 of the main text; www.inaturalist.org; Table S3). Figure S1. Records of free-ranging llamas in Monti del Chianti and Valdarno between 2017 and 2020. Figure S2. Some photos of llamas observed in 2017–2020 (cf. Table 1 of the main text; www.inaturalist.org; Table S3).
