# Supplementary material for: From the Andes to the Apennines: Rise and Fall of a Free-Ranging Population of Feral Llamas
Source: Animals (Basel). 2021 Mar 18;11(3):857. doi: 10.3390/ani11030857 (PMC8003056; doi:10.3390/ani11030857)
Supplement: Supplementary file 1 [file animals-11-00857-s001.pdf]

animals

MDPI

Article

From the Andes to the Apennines: rise and fall of a free-ranging population of feral llamas

Carlo Gargioni[1], Andrea Monaco[2], Gentile Francesco Ficetola[1], Lorenzo Lazzeri[3] and Emiliano Mori[4,*]

1. Department of Environmental Science and Policy, Università degli Studi di Milano, Via Celoria 26, 20133 Milan, Italy; carlo.gargioni@gmail.com; francesco.ficetola@unimi.it
2. ISPRA Institute for Environmental Protection and Research, Via Vitaliano Brancati 48, 00144 Rome, Italy; andrea.monaco@isprambiente.it
3. Research Unit of Behavioural Ecology, Ethology and Wildlife Management, Department of Life Sciences, University of Siena. Via P.A. Mattioli 4, 53100, Siena, Italy; lazzerilorenzo12@gmail.com. ORCID ID: 0000-0002-9556-6204
4. Istituto di Ricerca sugli Ecosistemi Terrestri (IRET), Consiglio Nazionale delle Ricerche (CNR), Via Madonna del Piano 10, 50019, Sesto Fiorentino (Florence), Italy; emiliano.mori@cnr.it. ORCID ID: 0000-0001-8108-7950.

Citation: Gargioni, C.; Monaco, A.; Ficetola, G.F.; Lazzeri, L.; Mori, E. From the Andes to the Apennines: rise and fall of a free-ranging population of feral llamas. *Animals* **2021**, *11*, 857. https://doi.org/10.3390/ani11030857

Academic Editor: Mathew Crowther and Koichi Kaji

Received: 9 February 2021
Accepted: 13 March 2021
Published: 18 March 2021

Publisher's Note: MDPI stays neutral with regard to jurisdictional claims in published maps and institutional affiliations.

Copyright: © 2021 by the authors. Submitted for possible open access publication under the terms and conditions of the Creative Commons Attribution (CC BY) license (http://creativecommons.org/licenses/by/4.0/).

Table S1. Questionnaire conducted to test for social perception of feral llamas in Central Italy. Responses 1 an 2 were considered as disagreement, responses 3 and 4 as agreement.

1) Are you aware of the population of free-ranging llamas occurring in this area? **YES□ NO□**
**How much do you agree with each of the following statements?** 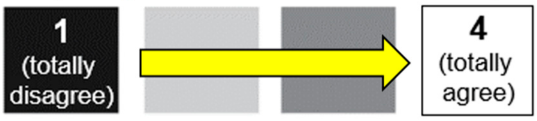
2) I appreciate the presence of llamas in this area
3) Llamas attract tourists
4) Remove llamas from the wild is useful to protect native fauna and flora
5) Remove llamas from the wild would affect local economy and human wellness

Table S2. Results of the standard questionnaire (N = 62 citizens, >18 years old, agreed to participate in this research). Agreement was expressed in a four-level scale ranging from 1 (totally disagree) to 4 (totally agree), as in Table S1.

ANSWERS (%) ↓	Appreciation of free-ranging llamas	Touristic attraction	Removal of llamas to protect native biodiversity	Llamas removal affects economy and wellness
**1**	14	35	40	81
**2**	19	14	13	6
**3**	40	32	29	13
**4**	27	19	18	0

Table S3. Results of media content analysis, with link to the analysed articles.

ID	Date	Journal	Journal name	Type of article	Experts	Webpage
1	01/03/2017	Local	Valdarno_Post	Positive	no	http://valdarnopost.it/news/lama-arriva-in-centro-a-panzano-in-chianti-probabilmente-un-esemplare-fuggito-dal-parco-di-cavriglia
2	01/03/2017	Local	Il_Sito_di_Firenze	Positive	no	https://www.ilsitodifirenze.it/content/786-lama-ferito-vaga-le-campagne-di-panzano-indagini-sulla-provenienza
3	01/03/2017	Local	Stamp_Toscana	Positive	no	https://www.stamptoscana.it/panzano-lama-ferito-sedato-e-curato-dal-servizio-veterinario/
4	01/03/2017	National	Il_Tirreno	Positive	no	https://iltirreno.gelocal.it/empoli/cronaca/2017/03/01/news/un-lama-ferito-recuperato-a-panzano-1.14959609
5	02/03/2017	Local	Gazzettino_del_Chianti	Positive	no	https://www.gazzettinodelchianti.it/greve-in-chianti/storia-lama-catturato-a-panzano/
6	06/09/2017	National	La_Repubblica	Positive	no	https://video.repubblica.it/edizione/firenze/chianti-i-lama-dell-ex-parco-zoo-di-cavriglia-vivono-in-liberta/283969/284582
7	09/12/2017	National	Il_Corriere_della_Sera	Neutral	no	https://corrierefiorentino.corriere.it/firenze/notizie/cronaca/17_dicembre_06/chianti-tra-vigneti-pascolano-lama
8	05/01/2018	National	La_Nazione	Neutral	no	https://www.lanazione.it/siena/cronaca/lama-chianti-1.3645097
9	05/01/2018	National	GoNews	Neutral	no	https://www.gonews.it/2018/01/05/lama-gaiole-in-chianti-animali/
10	10/05/2018	National	La_Nazione	Neutral	yes	https://www.lanazione.it/siena/cronaca/sorpresa-lama-chianti-monteluco-1.3902080
11	11/05/2018	National	Italia_Star_Magazine	Positive	no	https://www.italiastarmagazine.it/italia/lama-nel-chianti-e-tutto-vero-3373
12	21/07/2018	National	Il_Cittadino_Online	Positive	no	https://www.ilcittadinoonline.it/cronaca/provincia/si-vive-bene-nel-chianti-anche-lama-concordano/
13	23/08/2018	Local	SienaNews	Positive	no	https://www.sienanews.it/toscana/siena/i-lama-di-gaiole-in-chianti-mettono-su-famiglia-ecco-i-due-cuccioli/
14	03/11/2019	Local	QuiNews_Valdarno	Positive	no	https://www.quinewsvaldarno.it/montevarchi-in-valdarno-come-in-peru-lama-in-strada.htm
15	14/04/2020	Local	ArezzoNotizie	Negative	yes	https://www.arezzonotizie.it/attualita/animali-natura-lockdown-coronavirus-arezzo.html
16	12/05/2020	Local	SienaNews	Positive	no	https://www.sienanews.it/toscana/siena/gaiole-in-chianti-anche-i-lama-escono-dalla-quarantena/
17	08/08/2020	Local	SienaNews	Positive	no	https://www.sienanews.it/servizi/animali/ferito-uno-dei-lama-di-gaiole-in-chianti-il-video-e-lappello-di-una-famiglia-di-turisti/
18	08/08/2020	Local	ArezzoNotizie	Neutral	yes	https://www.arezzonotizie.it/attualita/lama-chianti-cavriglia-arezzo-firenze-toscana.html

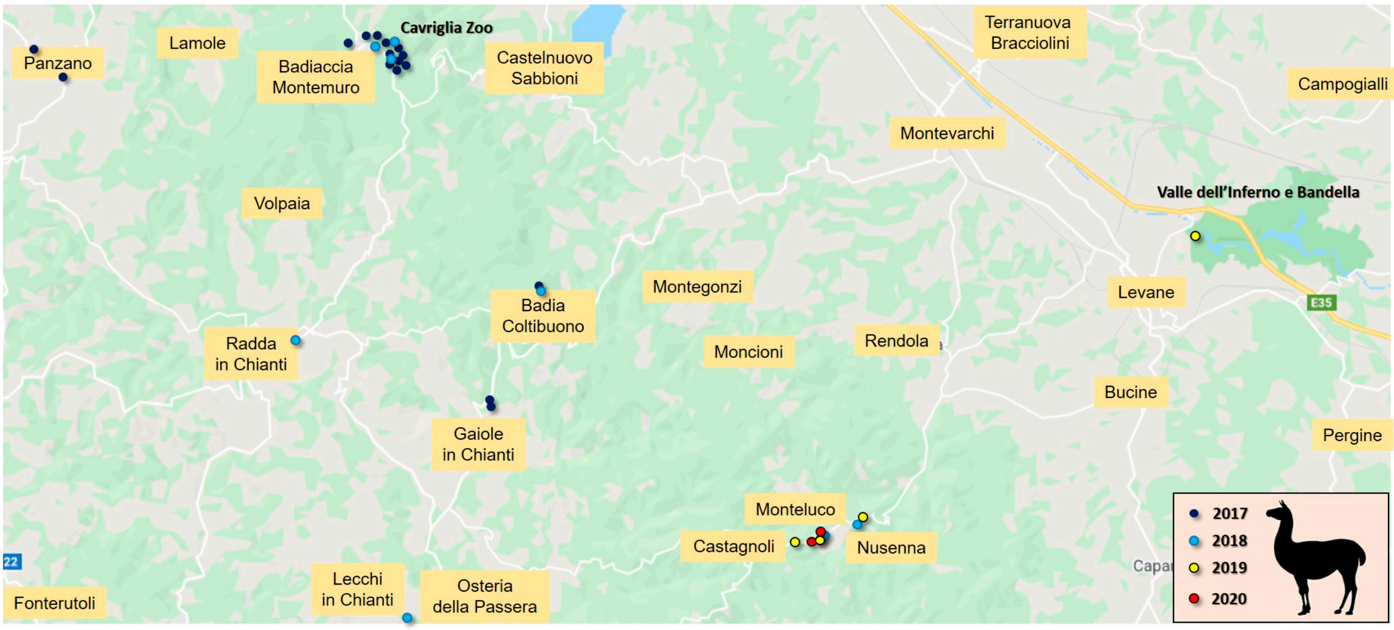

Figure S1. Records of free-ranging llamas in Monti del Chianti and Valdarno between 2017 and 2020.

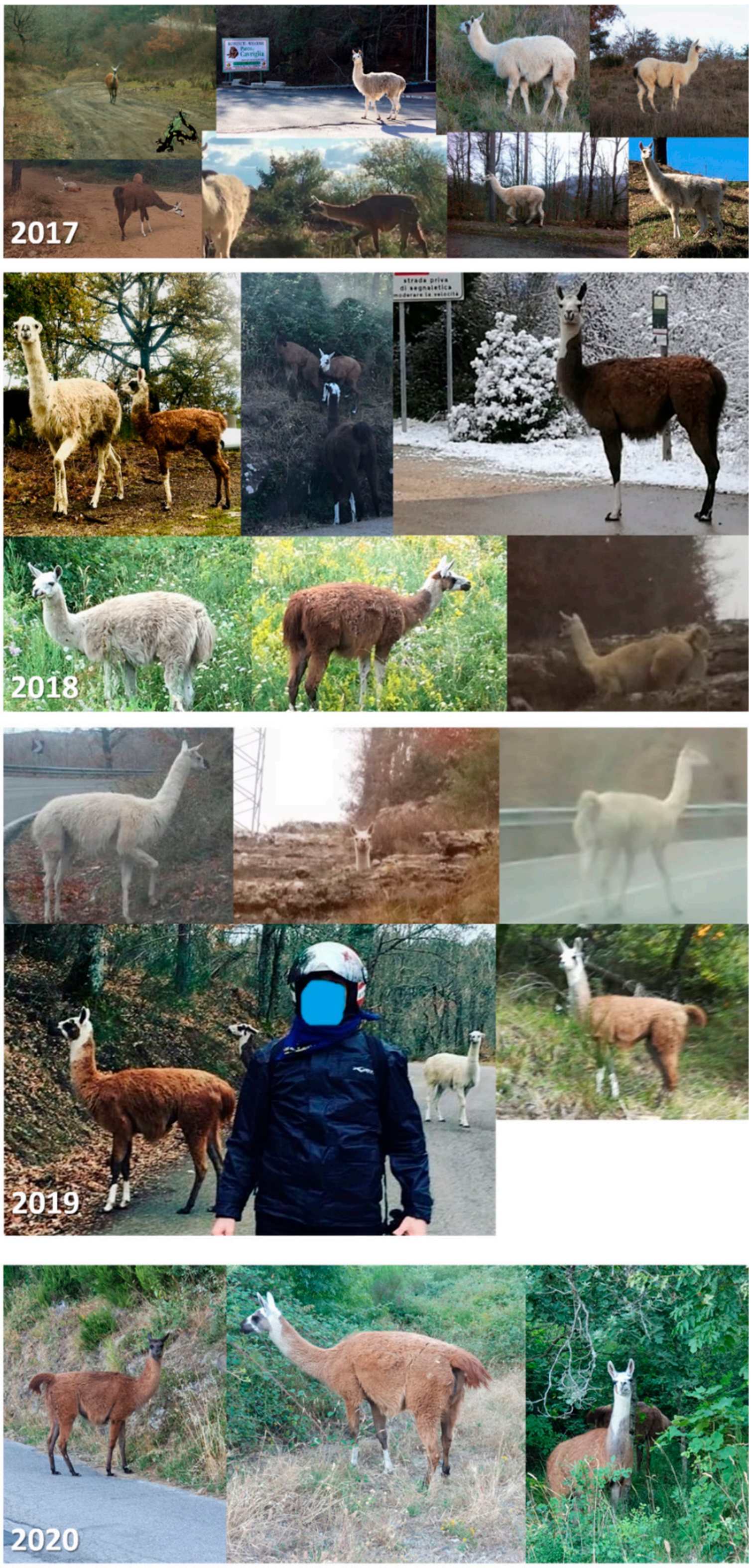

Figure S2. Some photos of llamas observed in 2017-2020 (cf. Table 1 of the main text; www.inaturalist.org; Table S3).
